# Short genome report of cellulose-producing commensal *Escherichia coli* 1094

**DOI:** 10.1186/s40793-018-0316-0

**Published:** 2018-05-09

**Authors:** Joaquin Bernal-Bayard, Laura Gomez-Valero, Aimee Wessel, Varun Khanna, Christiane Bouchier, Jean-Marc Ghigo

**Affiliations:** 1 Département de Microbiologie, Unité de Génétique des Biofilms, Institut Pasteur, 25-28 rue du Dr. Roux, F-75015 Paris, France; 2 Département de Génomes et Génétique, Unité de Biologie des Bactéries Intracellulaires, Institut Pasteur, 25-28 rue du Dr. Roux, F-75015 Paris, France; 3 Centre National de la Recherche Scientifique (CNRS). UMR 3525, 75724 Paris, France; 4 Institut Pasteur – Hub Bioinformatique et Biostatistique – C3BI, USR 3756 IP CNRS, Paris, France; 5 Institut Pasteur, Plate-forme Génomique, Pôle Biomics, CITECH 25-28 rue du Dr. Roux, F-75015 Paris, France

**Keywords:** *E. coli*, Commensal, Biofilm, Cellulose, Extracellular matrix, Bcs operon

## Abstract

Bacterial surface colonization and biofilm formation often rely on the production of an extracellular polymeric matrix that mediates cell-cell and cell-surface contacts. In *Escherichia coli* and many *Betaproteobacteria* and *Gammaproteobacteria* cellulose is often the main component of the extracellular matrix. Here we report the complete genome sequence of the cellulose producing strain *E. coli* 1094 and compare it with five other closely related genomes within *E. coli* phylogenetic group A. We present a comparative analysis of the regions encoding genes responsible for cellulose biosynthesis and discuss the changes that could have led to the loss of this important adaptive advantage in several *E. coli* strains.

Data deposition: The annotated genome sequence has been deposited at the European Nucleotide Archive under the accession number PRJEB21000.

## Introduction

Biofilms are ubiquitous microbial communities growing in close association with surfaces present in natural and anthropic environments [1]. Biofilm bacteria often self-assemble by producing a cohesive extracellular matrix that protects these multicellular aggregates against environmental changes and maintains the integrity of the biofilm structure [2]. Cellulose is a homo-polysaccharide composed of glucose units linked by β-1 → 4 glycosidic bonds and is the most common organic compound on Earth [3]. Initially studied in *Gluconacetobacter xylinus*, cellulose production is a widespread phenomenon shared by many commensal and pathogenic *Betaproteobacteria* and *Gammaproteobacteria*, including *Salmonella enterica* *serovar Typhimurium,* Klebsiella pneumoniae*,* *Burkholderia mallei**,* *Shigella boydii**,* *Yersinia enterocolitica**,* *Vibrio fischeri**,* *Pseudomonas putida* and many Escherichia coli strains [4, 5]. Here we report the complete genome sequence of E. coli 1094, a biofilm forming and cellulose-producing strain isolated from the feces of a healthy human male. E. coli 1094 lacks virulence factors commonly associated with pathogenic E. coli and is a member of E. coli phylogenetic group A [6, 7].

## Organism information

### Classification and features

*Escherichia coli* is a Gram-negative, rod-shaped, non-spore forming and facultative anaerobic species belonging to the *Enterobacteriaceae* family. They are commonly found in the intestines of endotherms and are taxonomically placed within the *Gammaproteobacteria* of the *Proteobacteria* phylum (Table 1).

**Table 1:** Classification and general features of *Escherichia coli* strain 1094

MIGS ID	Property	Term	Evidence code^a^
	Classification	Domain *Bacteria*	TAS [33]
		Phylum *Proteobacteria*	TAS [34]
		Class *Gammaproteobacteria*	TAS [34]
		Order “*Enterobacteriales”*	TAS [34, 35]
		Family *Enterobacteraceae*	TAS [36]
		Genus *Escherichia*	TAS [37, 38]
		Species *Escherichia coli*	TAS [37, 38]
		Type strain: *1094*	TAS
	Gram stain	Negative	IDA, TAS [39]
	Cell shape	Rod	IDA, TAS [39]
	Motility	Motile	IDA, TAS [39]
	Sporulation	Non	TAS [39]
	Temperature range	10 °C–45 °C	NAS
	Optimum Temperature	37 °C	IDA
	pH range; optimum	5.5–8.0; 7	
	Carbon source	Glucose	IDA
MIGS-6	Habitat	Human gut	
MIGS-6,3	Salinity	Not reported	
MIGS-22	Oxygen requirement	Facultative anaerobe	IDA, TAS [39, 40]
MIGS-15	Biotic relationship	Human specimen	NAS
MIGS-14	Pathogenicity	Nonpathogenic	NAS
MIGS-4	Geographic location	France	
MIGS-5	Sample collection	1999	
MIGS-4,1	Latitude	Not reported	
MIGS-4,2	Longitude	Not reported	
MIGS-4,4	Altitude	Not reported	

^a^Evidence codes - *TAS* Traceable Author Statement, *NAS* Non-traceable Author Statement, *IDA* Inferred from Direct Assay. These evidence codes are from the Gene Ontology project [12]

**Fig. 1:**
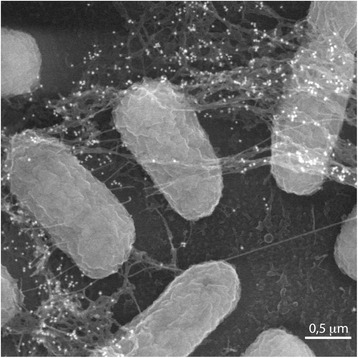
Scanning electron microscopy of cellulose producing strain *E. coli* 1094. *E. coli* 1094. Bacteria with cellulose labeled by CBM29–1-2 and immunogold antibodies. Scanning electron microscopy and immunogold labelling were performed at LBCME facility, Faculte de Medecine de Tours, Tours, France)

*E. coli* 1094 *is a commensal strain isolated from feces of a healthy human male.* Like many other natural *E. coli* isolates, 1094 produces cellulose as the main component of its biofilm extracellular polymeric matrix (Fig. 1) [8], and has been used as a model for studying both transcriptional regulation and function of cellulose biosynthesis genes (*bcs* genes) [8, 9], as well as to analyze the structure of the cellulose secretion machinery [10]. Here, we investigate global genomic differences between representative members of *E. coli* phylogenetic group A and discuss their functional consequences on cellulose production.

**Fig. 2:**
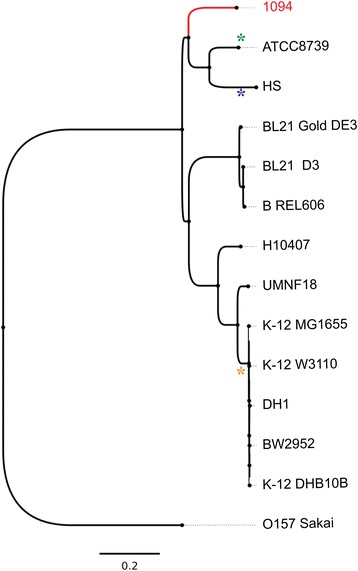
Phylogenomic comparison of *E. coli* strains from phylogenetic group A. The tree was inferred using Parsnp, a fast core-genome multi-aligner. Color-coded stars represent relevant genetic events related with the loss of cellulose production: orange, premature stop codon in *bcsQ*; purple, deletion of a large *bcs* region; green, premature stop codon in *bcsE*

*E. coli* *1094 was previously classified as a member of the phylogenetic group A based on* triplex PCR with a combination of primers amplifying two genes (*chuA* and *yjaA*), and an anonymous DNA fragment designated TSPE4.C2 [7]. Using all available complete genomes of phylogenetic group A, we performed a core genome alignment to produce a phylogenetic tree, and found it to be in agreement with previous phylogenetic classification [11], showing that *E. coli* strain 1094 is closely related to strains ATCC 8739 and HS (Fig. 2).

## Genome sequencing information

### Genome project history

*E. coli* 1094 has been used as a model to study different aspects of cellulose biosynthesis and biofilm formation [8, 9]. Cellulose production requires the expression of bacterial cellulose synthesis genes clustered in two divergent operons, as well as genes involved in general glucose metabolism [8], In order to further elucidate the genetic bases of cellulose synthesis, we chose to sequence *E. coli* 1094 using two approaches: Illumina and PacBio sequencing. While Illumina sequencing and subsequent downstream analysis generated 204 contigs, PacBio sequencing and assembly produced 4 contigs. A summary of the project information and its association with “Minimum Information about a Genome Sequence” [12] are provided in Table 2.

**Table 2:** Project information

MIGS ID	Property	Term
MIGS 31	Finishing quality	High quality drafts
MIGS-28	Libraries used	Two genomic libraries; one Illumina library, one PacBio standard library
MIGS 29	Sequencing platforms	Illumina Miseq2000, Pacific Biosciences RSII
MIGS 31.2	Fold coverage	354× Illumina, 114.3× PacBio
MIGS 30	Assemblers	CLC Bio (version 4.20), HGAP.3
MIGS 32	Gene calling method	GLIMMER2
	Locus Tag	EC1094V2
	Genbank ID	LT883139-LT883142
	GenBank Date of Release	05-DEC-2017
	GOLD ID	Gp0267270
	BIOPROJECT	PRJEB21000
MIGS 13	Source Material Identifier	CRBIP19.182
	Project relevance	Human commensal

### Growth conditions and genomic DNA preparation

*E. coli* 1094 was cultivated in LB medium overnight at 37 °C. High quality genomic DNA for sequencing was extracted using the Wizard Genomic DNA Kit (Promega) (for Illlumina approach), or the QiaAMP DNA Mini Kit (QIAGEN) (for PacBio approach).

### Genome sequencing and assembly

#### Illumina sequencing

Whole genome library preparation (NEXTflex PCR-Free DNA-Seq kit, Bioo Scientific) and sequencing following standard protocols developed by the supplier were performed at the Genomics platform at the Pasteur Institute. Single reads averaging 110 base pairs were collected on a HiSeq2000 (Illumina, San Diego, CA). Approximately 8,285,636 reads were assembled using CLC Bio (version 4.20) giving a total of 204 contigs. The final Illumina-based sequence includes 4,982,209 bases with a G + C content of 50.81%.

#### PacBio sequencing

Library preparation, sequencing, and assembly were performed by the Earlham Institute. PacBio sequencing libraries were prepared from 10 μg DNA using standard Pacific Biosciences protocols (Pacific Biosciences, Menlo Park, CA). Following construction, libraries were size selected, from 7 to 50 kb, using the Sage Science BluePippin™ system with a 0.75% gel cassette. Libraries were run on the Pacific Biosciences RSII sequencer at 350pM using P6/C4 chemistry. A Single Molecule Real Time (SMRT) cell was used, yielding 150,292 reads, and 1213 megabases of sequence data. Reads were assembled using PacBio’s hierarchical genome-assembly process (HGAP.3), with filtering and adaptor trimming performed as previously described [13]. The minimum seed read length used was 6 kb, with a quality score cutoff of 0.8. The Celera Assembler was used to produce 4 large contigs, using pre-assembled, error-corrected reads. The maximum contig length produced was 4,903,991 bases.

#### Illumina & PacBio comparison

**Fig. 3:**
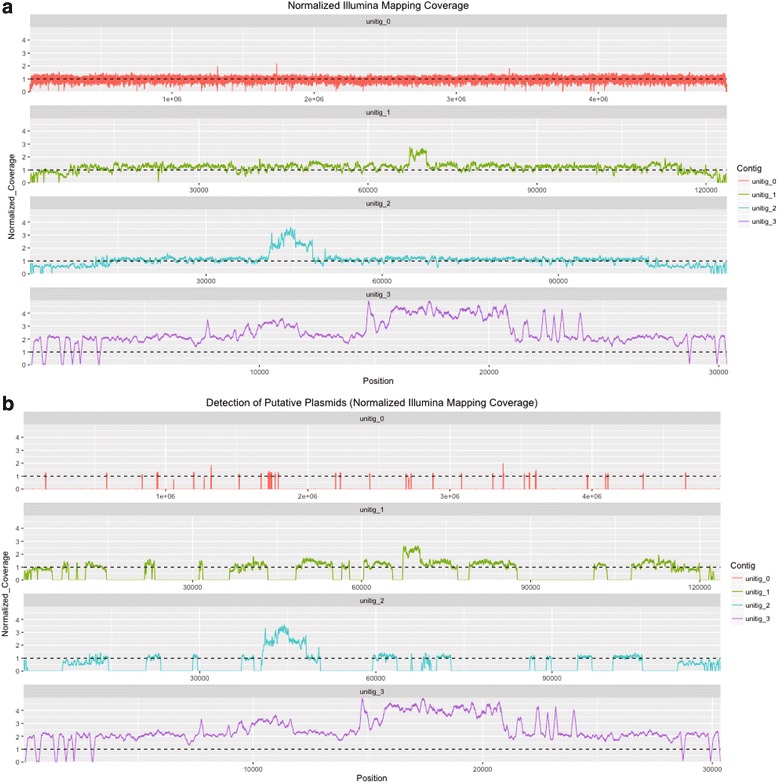
Illumina single-end reads mapped against PacBio contigs. **a** Copy number variation along the 4 PacBio large contigs, as determined from Illumina coverage. Copy numbers (ordinates) were normalized to 1 (for haploids). **b** Copy number variation along the 4 PacBio large contigs, as determined by remapping of putative plasmid reads identified by plasmidSPAdes. Copy numbers (ordinates) were normalized to 1 (for haploids)

Illumina single-end reads were mapped against the four large contigs generated by PacBio reads using the single-end mode of Bwa mem v0.7.4 [14] with default parameters (Fig. 3). Output SAM files were converted to BAM files using SAMtools v0.1.19 [15]. Sequencing coverage was computed using BEDtools v2.17.0 0 [16] and values were normalized to 1 (genomes are haploids), from the median coverage over the large contigs (354×). The mapping coverage along the four PacBio contigs validated the sequence assemblies. Some peaks of high coverage are observed in unitig_0, unitig_1 and unitig_2 (Fig. 3a), which suggests that multiple copies of these regions exist. By contrast, the coverage of the complete unitig_3 indicates that there is likely more than one copy per chromosome within each cell.

SPAdes v 3.9.1 [17, 18] was used for assembling Illumina reads, for detecting putative plasmids sequence with the options ‘--plasmid’, ‘-k 21,51,71’, ‘cut-off auto’ and ‘--careful’. Illumina reads were re-mapped onto the contigs using the single-end mode of Bwa mem v0.7.4 with default parameters. After converting output SAM files to BAM files by SAMtools, putative plasmid reads were extracted using SAMtools (option ‘view -F 4’) and recorded in a Fastq file by picardtools ‘SamToFastq’ [19]. Putative plasmid reads were mapped against the four large PacBio contigs using the single-end mode of Bwa mem v0.7.4 with default parameters. The coverage computed by BEDtools (Fig. 3b) indicates that the complete unitig_3 is classified as a putative plasmid sequence and also appears to exist in high copy number. For unitig_0, unitig_1 and unitig_2, the mapping coverage shows some portions of large contigs classified as putative plasmids. This may correspond to plasmids with similar coverage to the chromosome, due to low copy number, or to misclassification by plasmidSPAdes.

#### Sequence circularization

PacBio scaffold sequences were compared against themselves with the bl2seq BLASTN algorithm [20], and ACT [21] was used for synteny visualization. The resulting overlapping sequences were easily identified between the beginning and the end of each large contigs, suggesting that all four PacBio large contigs are circular. To determine the size of the chromosome and each plasmid, the size of the overlapping region (Unitig_0: 15,155; Unitig_1: 8,919; Unitig_2: 13,971; Unitig_3: 9,380) was subtracted from the length of each contig (Unitig_0: 4,903,991; Unitig_1: 123,705; Unitig_2: 118,720; Unitig_3: 30,364); the final sizes are reported in Table 3.

**Table 3:** Summary of 1094 genome: one chromosome and 3 putative plasmids

Label	Size (Mb)	Topology	INSDC identifier	RefSeq ID
Chromosome	4.888	Circular	LT883139	
Plasmid 1	0.115	Circular	LT883140	
Plasmid 2	0.105	Circular	LT883141	
Plasmid 3	0.021	Circular	LT883142	

### Genome annotation

The complete genome sequence was annotated with the RAST server [22] which predicted 5028 coding sequences and 110 RNAs.

## Genome properties

A summary of the genome of *E. coli* 1094 is included in Table 3. The genome statistics are provided in Table 4. Three putative plasmids were identified, and found to be circular. The genome of strain 1094 has a total length of 5,176,780 base pairs and a G + C content of 50.9%. The majority of the protein-coding genes were assigned a putative function (78.8%) while the remaining ones were annotated as hypothetical proteins. Genes in internal clusters were detected using BLASTclust with thresholds of 70% covered length and 30% sequence identity [23]. CRISPR, transmembrane helice, signalP and Pfam protein families predictions were done using CRISPRFinder [24], TMHMM Server v.2.0 [25], SignalP 4.0 [26] and Pfam 29.0 [27], respectively. The distribution of genes into COGs functional categories is presented in Table 5.

**Table 4:** Genome statistics

Attribute	Value	% of Total
Genome size (bp)	5,176,780	100.0
DNA coding (bp)	4,503,495	86.9
DNA G + C (bp)	2,340,599	50.9
DNA scaffolds	4	100.0
Total genes	5138	100.0
Protein coding genes	5028	97.8
RNA genes	110	2.1
Pseudo genes	0	0
Genes in internal clusters	232	4.5
Genes with function prediction	4390	85.4
Genes assigned to COGs	4827	93.9
Genes with Pfam domains	4377	85.2
Genes with signal peptides	398	7.7
Genes with transmembrane helices	1099	21.4
CRISPR repeats	11	0.2

**Table 5:** Number of genes associated with general COG functional categories

Code	Value	%age	Description
J	179	3.5	Translation, ribosomal structure and biogenesis
A	3	0.05	RNA processing and modification
K	315	6.3	Transcription
L	254	5.05	Replication, recombination and repair
B	0	0	Chromatin structure and dynamics
D	45	0.9	Cell cycle control, Cell division, chromosome partitioning
V	58	1.15	Defense mechanisms
T	159	3.16	Signal transduction mechanisms
M	285	5.66	Cell wall/membrane biogenesis
N	62	1.23	Cell motility
U	116	2.3	Intracellular trafficking and secretion
O	172	3.42	Posttranslational modification, protein turnover, chaperones
C	308	6.12	Energy production and conversion
G	771	15.3	Carbohydrate transport and metabolism
E	318	6.32	Amino acid transport and metabolism
F	109	2.16	Nucleotide transport and metabolism
H	136	2.7	Coenzyme transport and metabolism
I	139	2.76	Lipid transport and metabolism
P	280	5.56	Inorganic ion transport and metabolism
Q	67	1.33	Secondary metabolites biosynthesis, transport and catabolism
R	–	–	General function prediction only
S	1051	20.9	Function unknown
–	–	–	Not in COGs

The total is based on the total number of protein coding genes in the genome

## Insights from the genome sequence

*E. coli* 1094 sequence was aligned against selected genomes belonging to *E. coli*phylogenetic group A*.* Alignment and posterior NJ (neighbor joining) phylogenetic reconstruction was carried out with Parsnp, a fast core-genome multi-aligner, using default parameters [28]. Alignment and tree visualization was done with Gingr, a dynamic visual platform (Fig. 2) [28].

**Fig. 4:**
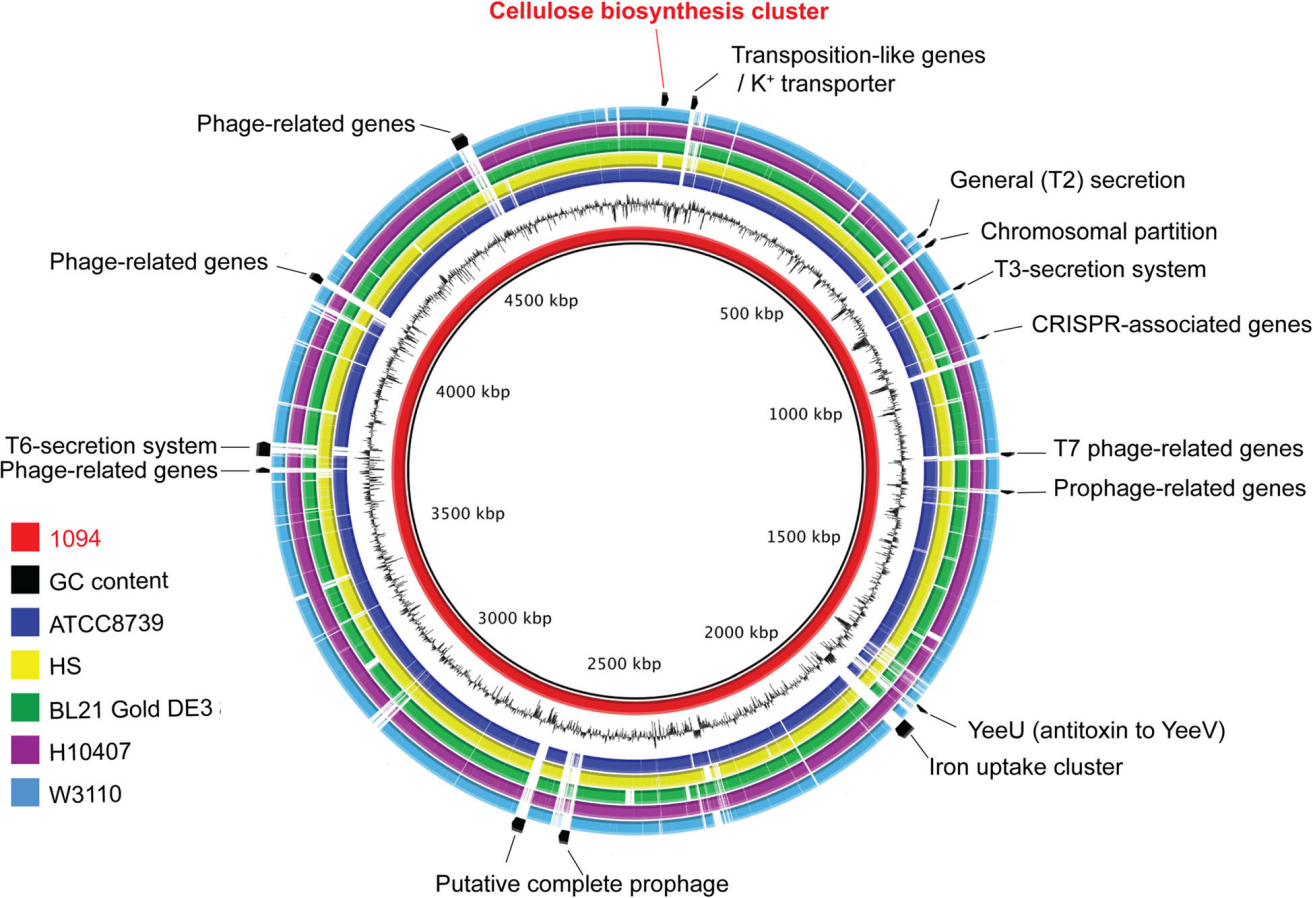
Circular representation of the *E. coli* 1094 genome. Genomic comparison of *E. coli* 1094 genome with the genomes of five other *E. coli* strains from phylogenetic group A. Each strain is color-coded (bottom-left). Relevant gene clusters and regions unique to *E. coli* 1094 and are indicated

We selected five *E. coli* genomes that are representative of the multiple clades within phylogenetic group A, and performed orthologous clustering to examine the genomic differences of *E. coli* 1094. [*E. coli* K12 W3110 (accession n°: AP009048), *E. coli* BL21-Gold(DE3)pLysS AG’ (accession CP001665), *E. coli* HS (accession n°: CP000802), *E. coli* ATCC 8739 (accession n°: CP000946) and the prototypical enterotoxigenic strain of *E. coli* ETEC H10407 (accession FN649414)] (Fig. 4). Our analysis reveals a core genome of 3409 genes shared among all strains (Fig. 4). *E. coli* ETEC H10407 possesses the highest number of specific genes (834), followed by *E. coli* 1094 (499), *E. coli* HS (440), *E. coli* BL21-Gold(DE3)pLysS AG’ (292), *E. coli* K12 W3110 (241) and *E. coli* ATCC8739 (216).

The analysis of the location of genes only present in *E. coli* *1094* identified eight 1094-specific regions, most of which are prophage or phage-associated proteins; two regions represent a putative complete prophage, containing genes encoding the phage capsid tail and replication proteins (Fig. 4). Other 1094-specific regions of interest contain CRISPR associated proteins. We also identified clusters only present in some, but not all *E. coli* strains analyzed, including (i) a putative complete type VI secretion system (present in 1094 and HS), (ii) a type III secretion system cluster (absent in BL21-Gold(DE3) and K12 W3110 strains of *E. coli*) (iii) a cluster of genes encoding invasins and an iron acquisition system, (present in 1094 and H10407), and (iv) several cellulose biosynthesis genes present in all strains, except for *E. coli* HS.

We performed a basic BLAST (BLASTN 2.6.1+, [29]) of each smaller contig identified, and report the following results. Unitig_1 shows sequence homology to *Salmonella enterica subsp. enterica* *serovar Senftenberg* strain 775 W plasmid *pSSE-**ATCC-43845* (Accession: CP016838.1), with 33% of the contig showing 99% identity. Unitig_2 displays sequence homology to *Klebsiella pneumoniae subsp. pneumoniae* strain 234–12 plasmid *pKpn23412–362*, with 39% of the contig showing 99% identity (Accession: CP011314.1); other regions of the contig show homology to multiple *E. coli* strains and plasmids. Unitig_3 displays sequence homology to *Citrobacter freundii* strain B38 plasmid pOZ182; 68% of the contig shows 96% identity (Accession: CP016765.1); in addition, two separate regions of 7.5 and 2.1 kb are highly homologous to *E. coli* plasmid pV004-a DNA and pV001-a DNA (Accession: LC056140.1 and LC056078.1). Taken together, this suggests that 1094 expresses 3 distinct circular plasmids.

### Comparative analysis of the bcs (bacterial cellulose synthesis) region

**Fig. 5:**
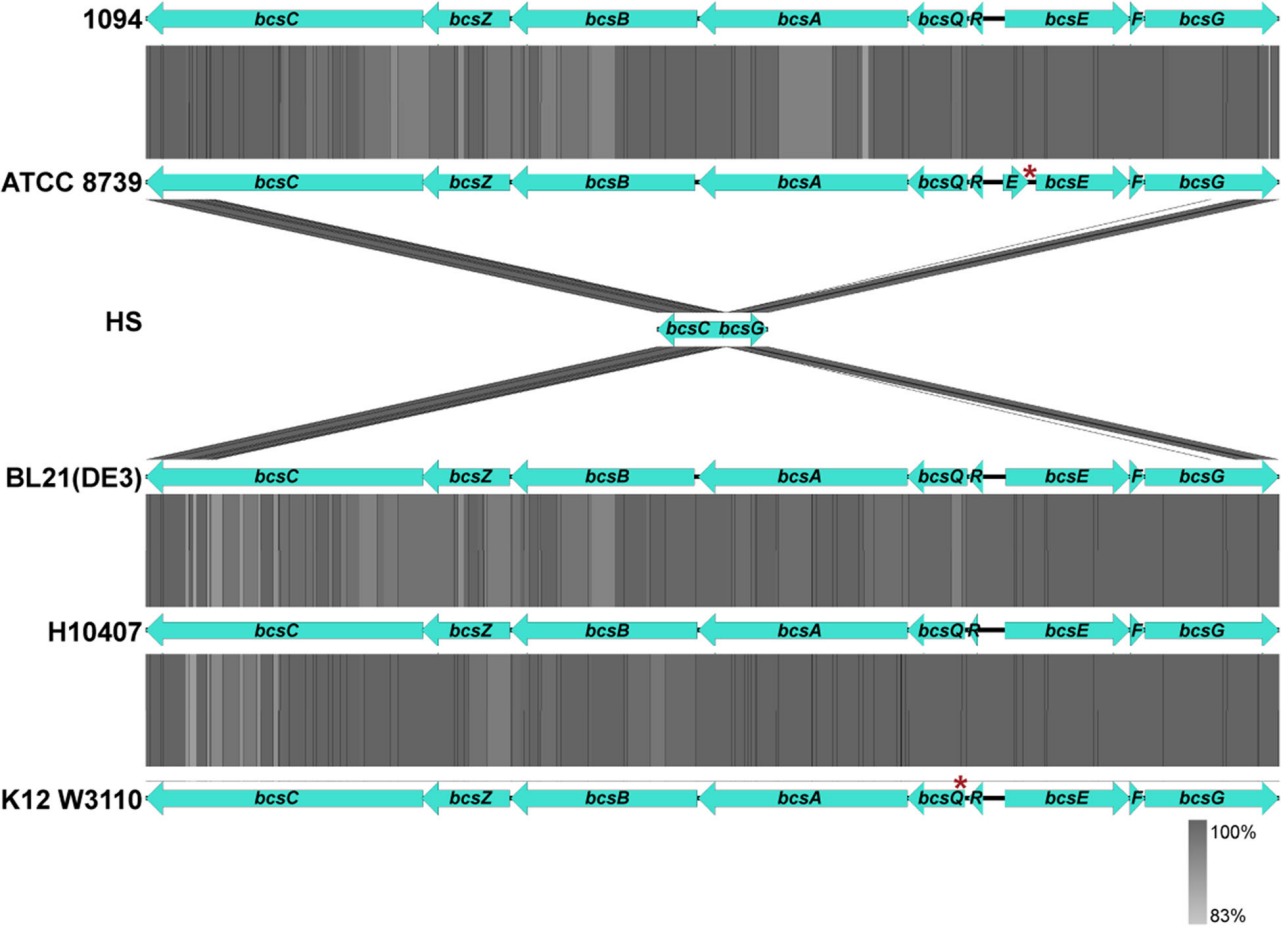
Genomic organization and Blastx comparison of the bacterial cellulose synthesis (*bcs*) region in six *E. coli* strains. The name of the corresponding genes are indicated. The gray color code represents the pairwise Blast matches between one strain, and the strain directly below it; darkness corresponds to higher similarity between the two strains, and strains were ordered based on phylogenetic relatedness. The star in red represents a SNP leading to a premature stop codon. The figure was generated using the Easyfig software [41]

We compared the region corresponding to the *E. coli* 1094 *bcs* operon with corresponding regions in the strains *E. coli* W3110*,* HS, BL21-Gold(DE3)pLysS AG’, ATCC 8739, and H10407, which are representative of phylogenetic group A. This analysis shows that whereas only partial fragments of the external genes of the *bcs* operon (*bcsC* and *bcsG*) exist in *E. coli* HS, the five other strains analyzed contain all genes within the *bcs* operon (Fig. 5). K12 derivative strains do not produce cellulose, as they contain a premature stop codon in the gene *bcsQ*, due to a single nucleotide polymorphism (SNP) in the region TTG/TAG (17 T > A) (Fig. 5) [30]. Serra et al., repaired this SNP, which resulted in cellulose production in *E. coli* K12 W3110, suggesting that the premature stop codon in *bcs*Q could either affect the function of BcsQ, or has a polar effect on neighboring genes [30]. The other SNPs (relative to *E. coli* 1094) observed in the *bcs* operons of *E. coli* K-12 strains result either in synonymous codons or in conservative amino acid exchanges. By contrast, comparison of the *bcs* operon sequence between *E. coli* 1094 and *E. coli* ATCC8739 revealed 100% sequence identity between *bcsQ*, *bcsR*, *bcsA* and *bcsB* genes. Finally, the sequence of the *bcs* operon in *E. coli* ATCC8739 contains one SNP in *bcsE* that leads to a premature stop codon, and may also have a polar effect on *bcsF* and *bcsG*, which are essential for cellulose production [8] (Fig. 5). *E. coli* strains BL21-Gold(DE3)pLysS AG’ and H10407 contain multiple SNPs in *bcs* genes that are essential for cellulose biosynthesis. Some of these SNPs lead to amino acid changes that could negatively affect the function of these proteins. *bcsA* is particularly interesting, as a pairwise genetic comparison in all six *E. coli* strains analyzed revealed the existence of multiple *bcsA* SNPs (between 21 and 29, depending on the strain pair compared). None of the SNPs resulted in amino acid exchange or a premature stop codon, suggesting a strong selection pressure for this specific amino acid composition. Our phylogenetic analysis of the phylogenetic group A allowed us to localize these evolutionary events, by following a parsimony criterion, which allowed us to infer that mutations leading to premature stop codons took place in two separate evolutionary events: the branch of the strain ATCC8739 and in the K12 common ancestor (Fig. 2). These mutations resulted in sequence interruption of *bcsE* and *bcsQ* respectively.

### Cellulose production of *E. coli* strains

**Fig. 6:**
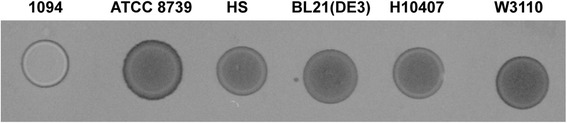
Cellulose production in six *E. coli* strains. Cellulose secretion was evaluated by the binding of calcofluor (CF), as visualized by colony fluorescence under ultraviolet light (fluorescence appears white in image). Five microliters of an overnight culture was spotted onto LB agar plates, containing 0.02% of CF and incubated for 24 h at 30 °C. Images were acquired using a G:BOX imaging system (Syngene, Frederick, MD)

Cellulose production was tested in the six *E. coli* strains analyzed in this study. We monitored colony fluorescence on LB-calcofluor plates, which is a common assay used to detect cellulose biosynthesis [31] (Fig. 6). This analysis revealed that only *E. coli* 1094 strain produced detectable levels of cellulose. As was previously shown, laboratory *E. coli* K12 strains do not produce cellulose [30]. Here we show that, as expected, *E. coli* HS is not able to produce this polymer, as it was known to lack all *bcs* genes. Interestingly, cellulose production was not detected in *E. coli* ATCC 8739, suggesting that the SNP in the region TGT/TGA (303 T > A) of *bcsE* described above could have a polar effect on the downstream genes. While the *bcsEFG* operon is essential for cellulose production [8], *bcsE* is only necessary for maximal cellulose production [32], suggesting that the inability to synthetize detectable levels of cellulose may be due to a polar effect of the *bcsEFG* operon. Finally, cellulose production was not detected in strains BL21-Gold(DE3)pLysS AG’ and H10407, which could be explained by the presence of multiple amino acid changes that potentially affect the biological activity of BcsB, BcsC and BcsQ proteins, which are essential for cellulose production.

## Conclusions

The human commensal intestinal isolate *Escherichia coli* strain 1094 naturally produces cellulose, a polysaccharide known to contribute to adhesion and biofilm development. We compared genomic content and cellulose production of closely related *E. coli* strains in phylogenetic group A, and found 1094 to be the sole strain to produce measureable levels of cellulose, and conclude that these strains lack the capacity to produce cellulose due to one or several SNPs in cellulose biosynthesis genes: non-synonymous SNPs in *bcsB*, *bcsC*, *bcsQ*, and a nonsense mutation in *bcsE*. The genome sequencing and annotation here provides valuable information for future study of the regulation of the *bcs* genes and cellulose production.

## Abbreviations

Bcs: Bacterial cellulose synthesis
CF: Calcofluor
RAST: Rapid Annotation using Subsystem Technology
SNPs: Single nucleotide polymorphism
